# Fostering Next-Gen Gerontologists: AI-Powered Research Training for Undergraduates

**DOI:** 10.1093/geroni/igaf122.3486

**Published:** 2025-12-31

**Authors:** Catheryn Koss

**Affiliations:** California State University, Sacramento, Sacramento, California, United States

## Abstract

Artificial intelligence (AI) is increasingly being integrated into academic and professional training as educators explore its potential to enhance student learning. By lowering barriers to entry, AI tools can help students who are intimidated by research gain hands-on experience in the research process, enabling them to produce high-quality, professional-level outputs such as research posters. This presentation will demonstrate how AI can be effectively incorporated into undergraduate curricula, drawing from experience using these tools in a research course focused on aging and the life course. Participants will learn practical strategies for leveraging different AI models to support student engagement in research, from understanding and evaluating academic literature to synthesizing key findings. Additionally, the session will showcase how AI tools, particularly ChatGPT, can assist students in conducting statistical analyses, interpreting results, and visualizing data through graph generation. By integrating AI into coursework, educators can help students develop critical thinking skills, improve their ability to navigate complex research, and gain confidence in quantitative methods. The presentation will include hands-on examples of AI applications in research assignments, discussions on ethical considerations, and best practices for balancing AI assistance with traditional learning methods. Attendees will leave with concrete strategies for integrating AI into their teaching to better prepare students for an evolving technological landscape in gerontology and related fields.

